# SARS-CoV-2 Infection-Associated Aortic Thrombosis Treated with Oral Factor Xa Inhibition

**DOI:** 10.1155/2022/7805900

**Published:** 2022-08-25

**Authors:** Alena Strýčková, Jakub Benko, Martin Jozef Péč, Monika Péčová, Jana Žolková, Monika Brunclíková, Tomáš Bolek, Ján Staško, Matej Samoš, Marián Mokáň

**Affiliations:** ^1^National Centre of Hemostasis and Thrombosis, Department of Hematology and Blood Transfusion, Jessenius Faculty of Medicine in Martin, Comenius University in Bratislava, Martin, Slovakia; ^2^Department of Internal Medicine I, Jessenius Faculty of Medicine in Martin, Comenius University in Bratislava, Martin, Slovakia

## Abstract

Coronavirus disease 2019 (COVID-19) is an acute complex systemic disorder caused by the severe acute respiratory syndrome coronavirus 2 (SARS-CoV-2).While SARS-CoV-2 is known to cause significant pulmonary disease, various extrapulmonary manifestations of COVID-19 have also been reported. Growing evidence suggests that COVID-19 is associated with coagulopathy leading to micro and macrovascular complications. Although in patients with COVID-19, venous thromboembolic events are more frequent, arterial thrombosis also occurs at an increased rate. These often lead to acute life-threatening ischemia, which requires urgent diagnosis and treatment. We present case reports of two patients with an abnormal thrombus formation in the thoracic aorta who recently overcame COVID-19, which led to systemic embolism and splenic infarction. Ambulatory oral factor Xa inhibitor therapy led to aortic thrombosis resolution in both patients.

## 1. Introduction

Coronavirus disease 2019 (COVID-19), an acute complex systemic disorder, is caused by the severe acute respiratory syndrome coronavirus 2 (SARS-CoV-2). COVID-19 causes a spectrum of disorders, with various clinical manifestations. Patient condition might vary from asymptomatic or mildly symptomatic respiratory infection to severe interstitial pneumonia progressing to acute respiratory distress syndrome (ARDS), sepsis, and multiorgan failure [1, 2]. Some patients develop a severe proinflammatory state associated with a unique coagulopathy and procoagulant endothelial phenotype. Several changes in hemostasis, such as prolongation of prothrombin time, elevated levels of D-dimer, fibrinogen, factor VIII and von Willebrand factor, and low platelet count, have been previously reported in patients with COVID-19 [2, 3]. In addition, the disease was repeatedly associated with higher risk of venous thromboembolism (VTE) in hospitalized patients [3]. Several studies reported a VTE incidence of 2–5% [4]. Nevertheless, patients with COVID-19 could be at increased risk for arterial thrombosis due to suggested COVID-19-associated endothelial damage, usually manifesting in individuals without significant preexisting atherosclerosis [2, 5, 6]. We present a case series of patients with acute aortic thrombosis manifesting with systemic embolism associated with recent COVID-19 infection. Ambulatory oral factor Xa inhibition (FXaI) was used for the treatment of thromboembolic events.

## 2. Case Reports

### 2.1. Case No. 1

**Figure 1:**
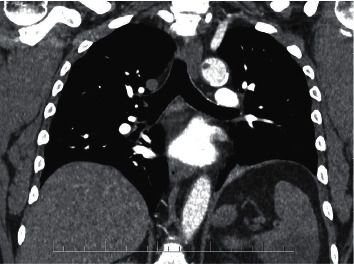
CT angiography showing aortic thrombosis (case no. 1).

A 58-year-old male patient was admitted to our department for left subcostal pain. The patient had a history of symptomatic COVID-19 disease one month prior the hospitalization, leading symptoms were an elevated body temperature of up to 38°C lasting three days and weakness. The patient had a history of arterial hypertension and was treated with perindopril, amlodipine, and indapamide. Laboratory tests showed an elevated C-reactive protein (CRP) levels, creatinine levels, elevated concentration of D-dimer (3.86 g/mL), and fibrinogen (12.06 g/L). Leukocytosis with neutrophilia was present in the blood count. Other coagulation parameters were normal. Microbiological examinations were negative. A computed tomography pulmonary angiogram (CTPA) was performed without signs of pulmonary embolism. An accessory finding of a mural thrombus on the posterior wall of the descending aorta just behind the subclavian artery was found (Figure 1), together with a finding of a splenic infarction. A regular clinical examination for malignancy was performed, but no signs of cancer were found. No other risk factors for arterial or venous thrombosis were found. We examined antiphospholipid and anticardiolipin antibodies to exclude antiphospholipid syndrome with negative results. We started anticoagulation therapy immediately (nadroparin dosed 9500 IU twice daily subcutaneously for initial 5 days, then apixaban dosed 5 mg twice daily). Blood cultures were negative; thoracic and transesophageal echocardiography was performed, and no cardiac source of embolism was found. The further course of hospitalization went uncomplicated. The patient was discharged home on oral apixaban with an outpatient follow-up. Two months after the discharge, a control CT angiography of the aorta was performed. There was no residual thrombosis in the descending aorta. During this follow-up period, the white blood cells count restored to normal values. Based on these results, anticoagulation was subsequently terminated.

### 2.2. Case No. 2

**Figure 2:**
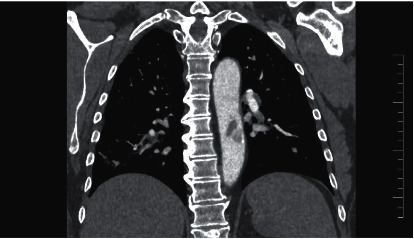
Fluttering aortic thrombosis on CT angiography (case no. 2).

A 62-year-old female patient with a history of arterial hypertension, gastroesophageal reflux disease, autoimmune thyroiditis, and recent COVID-19 infection presented with sudden pain and paresthesia in the left lower extremity. Laboratory tests showed slightly elevated CRP (21.6 mg/L) and elevated concentration of D-dimer (1.2 g/mL) and fibrinogen (4.45 g/L). Other coagulation parameters were normal. Antiphospholipid and anticardiolipin antibodies were negative. The patient overcame COVID-19 infection a month before, with bilateral pneumonia requiring hospitalization and oxygen supplementation. CT angiography was performed showing a fluttering thrombus in the descending aorta (Figure 2) and a closure of the left popliteal artery. The patient had no history of arterial or venous thrombosis, and evaluation for malignancy was also negative. The popliteal artery thrombotic closure was confirmed by digital subtraction angiography (DSA), and local thrombolysis was administrated, followed by intravenous body weight-adjusted heparin administration. Control DSA showed successful recanalization of the occluded popliteal artery. The patient was discharged home on oral rivaroxaban (20 mg daily) and aspirin (100 mg daily). A month later, a control CTA of the thoracic and abdominal aorta was performed with no signs of thrombosis.

## 3. Discussion

COVID-19 may predispose patients to an increased risk of thrombotic complications through various pathophysiological mechanisms [7]. Although venous thrombotic events in COVID-19 patients have been well described, there is limited evidence about arterial thrombosis in patients with COVID-19 [8]. According to the study of Cheruiyot et al., the anatomical distribution of arterial thrombotic events occurred in limb arteries (39%), cerebral arteries (24%), great vessels (aorta, common iliac artery, common carotid artery, and brachiocephalic trunk; 19%), coronary arteries (9%), and superior mesenteric artery (8%) [8]. In our cases, patients with initial abnormal thrombus formation in thoracic aorta manifested with secondary systemic embolism (with splenic infarction and acute limb ischemia). Therefore, CT-aortography should be probably included in the diagnostic approaches when searching for “cardiac source” of systemic embolism in patients with COVID-19. In both patients, COVID-19 was confirmed by a reverse transcription-polymerase chain reaction (PCR). Both patients overcame COVID-19 approximately one month before the diagnosis of aortic thrombosis. Whereas one patient had a mild course of the disease, the second patient had a moderate course of the disease with the need for hospitalization and oxygen administration. This indicates that there is no direct relationship between the risk of COVID-19-associated arterial thrombosis and COVID-19 clinical severity.

Another, yet not answered, question is how to treat COVID-19-related arterial thrombosis and systemic embolism. Therapeutic anticoagulation is being traditionally administrated in acute phase, preferring parenteral heparin or low-molecular-weight heparin (LMWH) administration in COVID-19 patients. Systemic thrombolysis could be an option in severe cases with life-threatening acute ischemia [9]; however, systemic thrombolysis is connected with high risk of serious bleeding. Thus, local thrombolysis could be a favorable approach, allowing the reduction of drug doses and reducing the risk of hemorrhage [10]. In our best knowledge, one of our cases is just the second one reporting the successful use of local (catheter-derived) thrombolysis in patient with COVID-19-related acute limb ischemia. Going further, LMWH anticoagulation is traditionally preferred in individuals with COVID-19. However, long-term LMWH therapy is unpleasant, connected with local complications, which reduce the compliance and possibly lead to increased risk of therapy failure. Oral direct FXaI could be a useful alternative therapeutic option, as it demonstrates the therapeutic effect in both arterial and venous thrombosis [11]. Nevertheless, the real-world experiences with this therapy in patients with COVID-19-related arterial thrombosis/systemic embolism are very limited. In our best knowledge, this is the first report about successful use of ambulatory oral FXaI inhibition for the treatment of COVID-19-related aortic thrombosis and systemic embolism. Furthermore, it is not entirely clear whether to coadminister antiplatelet therapy in these patients or not. Searching the relevant medical literature, there is no study dealing with this problem. Right now, the issue remains unexplained, and it is probably necessary to use a “patient-tailored” approach and assess the risk of future atherosclerosis-related vascular events. Concluding, the issue of optimal antithrombotic therapy for arterial thrombosis and systemic requires further research.

## Data Availability

The data used to support this study are available from the corresponding author upon request.
